# Mental Distress Among Norwegian Adults During the COVID-19 Pandemic: Predictors in Initial Response and Subsequent Trajectories

**DOI:** 10.3389/ijph.2023.1606164

**Published:** 2023-10-25

**Authors:** Li Lu, Laurie J. Hannigan, Ragnhild E. Brandlistuen, Ragnar Nesvåg, Lill Trogstad, Per Magnus, Anna Bára Unnarsdóttir, Unnur A. Valdimarsdóttir, Ole A. Andreassen, Helga Ask

**Affiliations:** ^1^ Institute of Health Management and Policy, School of Public Policy and Administration, Xi’an Jiaotong University, Xi’an, China; ^2^ Norment Centre, Institute of Clinical Medicine, University of Oslo, Oslo, Norway; ^3^ Department of Mental Disorders, Norwegian Institute of Public Health, Oslo, Norway; ^4^ Nic Waals Institute, Lovisenberg Diaconal Hospital, Oslo, Norway; ^5^ Population Health Sciences, Bristol Medical School, University of Bristol, Bristol, United Kingdom; ^6^ Department of Child Health and Development, Norwegian Institute of Public Health, Oslo, Norway; ^7^ The Norwegian Mother, Father and Child Cohort Study (MoBa), Norwegian Institute of Public Health, Oslo, Norway; ^8^ Division of Infection Control, Norwegian Institute of Public Health, Oslo, Norway; ^9^ Centre for Fertility and Health, Norwegian Institute of Public Health, Oslo, Norway; ^10^ Centre of Public Health Sciences, Faculty of Medicine, School of Health Sciences, University of Iceland, Reykjavik, Iceland; ^11^ Unit of Integrative Epidemiology, Department of Environmental Medicine (IMM), Karolinska Institutet, Stockholm, Sweden; ^12^ Department of Epidemiology, Harvard TH Chan School of Public Health, Boston, MA, United States; ^13^ Norment Centre, Division of Mental Health and Addiction, Oslo University Hospital, Oslo, Norway; ^14^ Promenta Research Center, Department of Psychology, University of Oslo, Oslo, Norway

**Keywords:** COVID-19, quarantine, mental distress trajectory, latent growth modelling, MoBa

## Abstract

**Objectives:** To identify factors associated with change in mental distress at the onset of the COVID-19 pandemic, relative to pre-pandemic levels, and with changes during the following 1.5 years.

**Methods:** The prospective Norwegian Mother, Father and Child Cohort Study collected eight waves of data during the pandemic (March 2020–September 2021) in 105,972 adult participants used for this analyses. A piecewise latent growth model was fitted to identify initial level and longitudinal changes in mental distress.

**Results:** Mental distress peaked at the beginning of the pandemic. Factors associated with initial increases were: medical conditions, living alone, history of psychiatric disorders, lower education, female sex, younger age, and obesity. Being quarantined or infected with SARS-CoV-2 were associated with increasing distress while being vaccinated was associated with reduced mental distress.

**Conclusion:** Having a chronic disease and being quarantined or infected by the SARS-CoV-2 virus were associated with more mental distress during the pandemic. This knowledge is important for planning interventions to support individuals during future pandemics and other societal crises.

## Introduction

Being infected with severe acute respiratory syndrome coronavirus 2 (SARS-CoV-2) has been associated with worsened mental health, particularly for the most severely infected (i.e., being hospitalized or bedridden for several days) [1–3]. Declining mental health in the population during the pandemic, regardless of infection status, has also been widely reported; including increases in symptoms of anxiety [4–8], depression [4, 6–8], elevated psychological distress [3, 9–11], sleep problems [5, 6], loneliness [11, 12], and COVID-19 related fears [13]. Social and environmental changes necessitated by the pandemic and often imposed by governments might have played a direct or indirect role in driving these changes [14]. With much of the research into the relative importance of SARS-CoV-2 infection and public health measures on mental health during the pandemic being cross-sectional in design and based on convenience samples, data from population-based, prospective cohorts are urgently needed.

Identifying factors associated with vulnerability and resilience trajectories of mental distress during the COVID-19 pandemic are key for planning targeted interventions to reduce the negative mental health impact of future pandemics and health crises. However, since most existing studies lack data on pre-pandemic mental health, there is no way to ascertain how specific the vulnerability or resilience-associated factors they identify are to mental health changes associated with the pandemic; nor to rule out reverse causation. One study showed that female gender, young age, lower income and educational attainment, living alone, and having pre-existing mental health conditions were risk factors for anxiety and depression at the start of lockdown, differences were still evident 20 weeks later [8]. Yet, the study lacked comparable pre-pandemic data, meaning that it was possible that these factors were generally associated with mental health changes across time, and not specifically predictive of changes in the context of the pandemic. The use of large population-based longitudinal data, with long follow-up periods, and rich individual-level information on pre-pandemic mental health can help address this problem.

Using the prospectively and contemporaneously collected data from over 100,000 participants of the Norwegian Mother, Father and Child Cohort Study (MoBa), the first aim of the current study was to estimate initial and longitudinal changes in mental distress in the Norwegian adult population during the COVID-19 pandemic, from pandemic onset until mass COVID-19 vaccination. During this time, the Norwegian government issued several orders and restrictions to alleviate the COVID-19 pandemic, such as closure of schools, remote work, stricter border controls, etc [15]. The second aim of the current study was to identify factors that put individuals at increased risk of worse trajectories of mental health during the pandemic. Crucially, with adjustment for pre-pandemic levels of mental distress based on the same measures of symptomatology, we could estimate the role of other predictors on mental distress trajectories during the pandemic while controlling for individuals’ average levels of pre-pandemic mental distress.

Based on the existing literature [8, 16], we hypothesized that level of mental distress would be elevated at the initial stage of the pandemic, with recovery over time, and pandemic exposures such as income loss, SARS-CoV-2 infections, or quarantine experience would be associated with increases in the level of mental distress experienced during the pandemic.

## Methods

MoBa is an ongoing, population-based pregnancy cohort study conducted by the Norwegian Institute of Public Health [17, 18]. Participants were recruited from all over Norway from 1999 to 2009. The women consented to participation in 41% of the pregnancies. The cohort now includes 114,500 children, 95,200 mothers and 75,200 fathers. The establishment of MoBa was based on a license from the Norwegian Data Protection Agency and approval from The Regional Committees for Medical and Health Research Ethics. The MoBa cohort is now based on regulations related to the Norwegian Health Registry Act. The current study was approved by The Regional Committees for Medical and Health Research Ethics (14140). All survey participants provided informed consent. In the current study we use pre-pandemic data from version 12 of the quality-assured data files released for research in January 2019.

**FIGURE 1:**
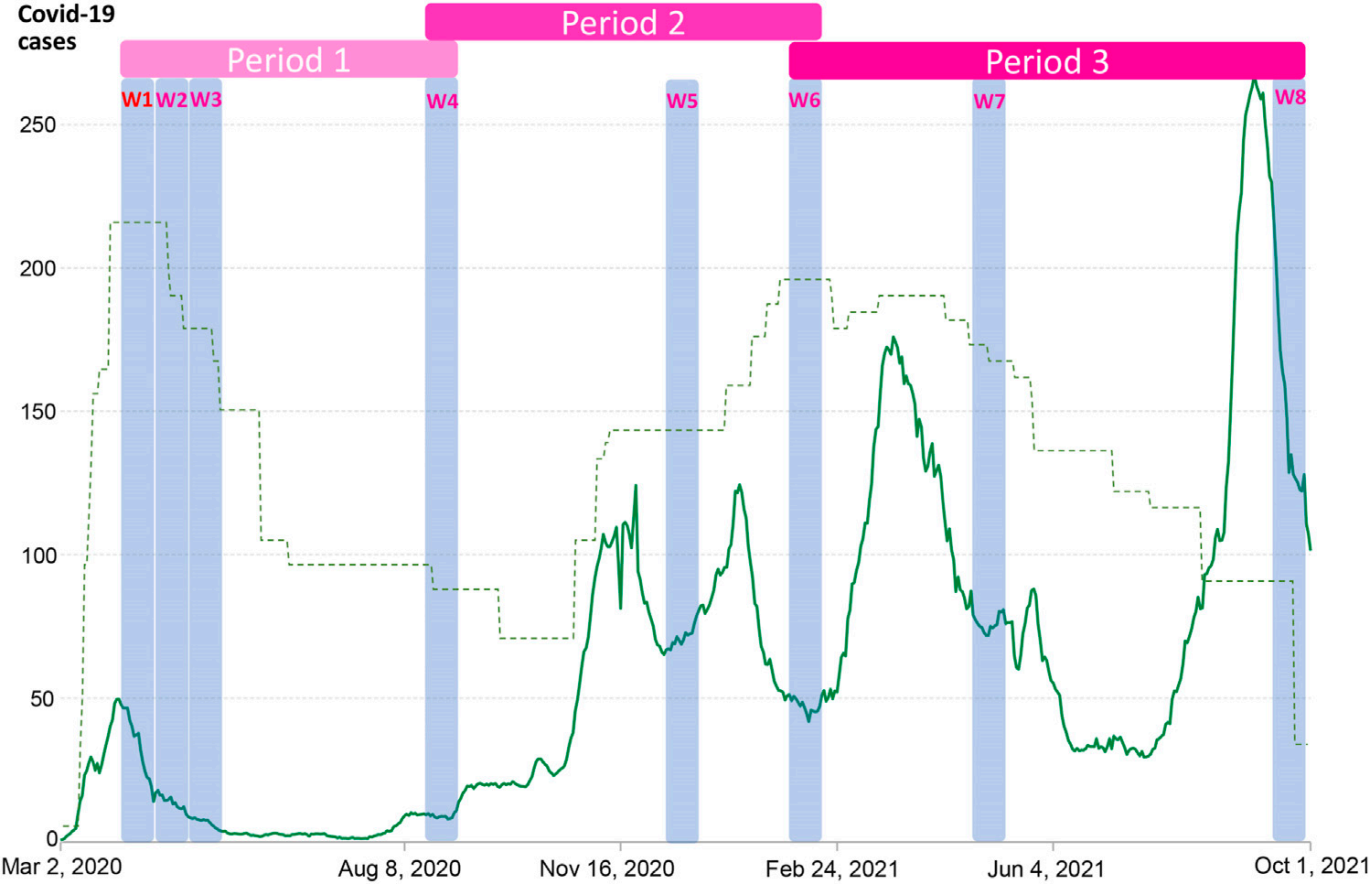
Timing of MoBa COVID-19 data collections (W1–W8) including Hopkins Symptom Checklist-5 (Oslo, Norway. 2023). Date of each wave (data collection): *Wave 1*, 31 March–14 April 2020; *Wave 2*, 14–29 April 2020; *Wave 3*, 29 April–12 May 2020; *Wave 4*, 19 August–1 September 2020; *Wave 5*, 8–21 December 2020; *Wave 6*, 2–17 February 2021; *Wave 7*, 28 April–11 May 2021; *Wave 8*, 16–29 September 2021. Notes: The green line shows daily new confirmed COVID-19 cases in Norway per million people. The dotted line indicates COVID-19 Stringency Index—a composite measure based on nine response indicators including school closures, workplace closures and travel bans (unscaled, but show the relative stringency across time periods). Source: Oxford COVID-19 Government Response Tracker, Blavatnik School of Government, University of Oxford. OurWorldInData.org/coronavirus. Periods 1/2/3 are defined according to the general trend in restrictions and in order that each period contains at least three waves of data collection for modelling of linear slopes—see the Supplementary Material for further details.

From March 2020, web-based questionnaires were sent to all adult MoBa participants biweekly to collect Covid-19-related information, notedly, the questionnaires were general that included questions on general health, infections, and vaccination, etc., thus the willingness to answer the questionnaires were not likely to be driven by present or former mental health issues. Participants reported their mental distress in eight waves of data collection. In Figure 1, data collection time points are plotted together with the national level of mitigating strategies and the average daily number of new cases of SARS-CoV-2 during the first 1.5 years of the pandemic. Wave 1 to 8 were respectively responded to from 31 March to 14 April 2020, 14–29 April 2020, 29 April–12 May 2020, 19 August–1 September 2020, 8–21 December 2020, 2–17 February, 2021, 28 April–11 May 2021, and 16–29 September 2021. Details of each data collection (wave) during the pandemic included in this study are described in the Supplementary Material. We included participants with data from at least three of the eight waves of COVID-19 data collections (*N* = 105,972).

### Measures

#### Outcomes

*Mental distress* was measured using the 5-item version of the Hopkins Symptoms Checklist (HSCL-5), where participants report their experience of two symptoms of anxiety and three symptoms of depression during the past 2 weeks [19]. Each item has four response options, ranging from “not at all” [1] to “extremely” [4]. HSCL-5 has been widely used and is validated with good psychometric properties in the Norwegian population (Cronbach’s α: 0.87) and in other countries [20, 21]. We excluded records with two or more missing items.

#### Covariates

We used the mean of two previous HSCL-5 measures as an indicator of *average levels of mental distress across pre-pandemic years*. These included responses on the first available measure, collected between October 1999 and July 2009 (in the mother’s 15th week of gestation) and on the most recent measure before the pandemic (collected when the children were 8 years for the mothers and in year 2015 for the fathers). If data for one measure point was missing, we used the score on the other as indicator of pre-pandemic mental distress.

*Baseline sociodemographic characteristics* included sex, age, education level, and living condition in Wave 1, i.e., living alone. *Health factors* included current Body Mass Index (BMI), which was assessed during the pandemic; history of psychiatric disorders; and chronic medical conditions, that were reported in Wave 1–3. *COVID-19-related variables* included income loss, determined by the question included in data collections during the two first time periods; SARS-CoV-2 infection, measured in all waves; and being in quarantine. Information concerning vaccination (SARS-CoV-2) status was available in the surveys Wave 6–8. Details of the construction of each variable are reported in the Supplementary Material.

### Statistical Analyses

Piecewise latent growth modelling based on data from the eight waves during the pandemic was performed to allow for a nonlinear pattern across time. As a result, our model has one intercept and three slopes, one for each period (see Figure 1 and model illustration in Supplementary Figure S1). Individual characteristics and health factors were included as predictors of variation in the intercept and all slopes in a single model, equivalent to a multiple regression. In addition, the period-specific COVID-19-related variables were included as predictors for relevant slopes. We ran our model both with and without adjusting for pre-pandemic mental distress, to investigate which predictors were general rather than specific to mental health changes in a pandemic context. All data analyses were performed using *lavaan* version 0.6-9 [22] in R version 4.0.0 [23] via RStudio [24]. Full Information Maximum Likelihood estimation was used to handle the missing values [25]. We used False discovery rate (FDR) to correct for multiple testing.

## Results

Descriptive statistics are presented in Table 1. The mean mental distress level was highest at the initial stage of the pandemic (mean = 7.08, 95% CI = 7.06–7.10), and higher than pre-pandemic mental distress (6.09, 95% CI = 6.08–6.11). There was an overall pattern of decreasing symptoms of mental distress during the first period of the pandemic, increasing symptoms during the second period, and decreasing symptoms in the final period (Supplementary Figure S2). Inter-individual variability in mental distress trajectories was substantial, with significant variance terms for each of the growth factors. The model fit was relatively good (i.e., with a root mean square error of approximation (RMSEA) < 0.06 and comparative fit index (CFI) > 0.95) [26].

**TABLE 1:** Descriptive characteristics for included sample (*N* = 105,972) (Oslo, Norway. 2023).

Variables		*n*	%^a^	Mean (se)
Pre-pandemic mental distress		101,982	96.2	6.09 (0.01)
Mental distress in	Wave 1	99,851	94.2	7.08 (0.01)
	Wave 2	99,640	94.0	6.92 (0.01)
	Wave 3	97,189	91.7	6.78 (0.01)
	Wave 4	60,736	57.3	6.34 (0.01)
	Wave 5	66,774	63.0	6.97 (0.01)
	Wave 6	82,374	77.7	6.73 (0.01)
	Wave 7	75,670	71.4	6.59 (0.01)
	Wave 8	72,447	68.4	6.26 (0.01)
Sex	Male	42,800	40.4	
	Female	63,172	59.6	
Age group (years)	25–34	829	0.8	
	35–44	36,433	34.4	
	45–54	61,653	58.2	
	55–64	6,594	6.2	
	65 or more	314	0.3	
	Missing	149	0.1	
Education	Master or PhD	30,610	28.9	
	Bachelor’s/University degree	37,123	35.0	
	Upper secondary, vocational or other	30,846	29.1	
	Compulsory	2,230	2.1	
	Missing	5,163	4.9	
BMI	<25 (Underweight or normal)	33,978	32.1	
	25–30 (Overweight)	29,358	27.7	
	>30 (Obese)	13,433	12.7	
	Missing	29,203	27.6	
History of psychiatric disorders	Yes	16,017	15.1	
N chronic medical conditions	1	17,315	16.3	
	2	2,428	2.3	
	>2	447	0.4	
Living alone during the pandemic	No	98,672	93.1	
	Yes	1,114	1.1	
	Missing	6,186	5.8	
Pandemic-specific measures during the first period				
Income loss	No	83,394	78.7	
	Yes, some loss	14,885	14.0	
	Yes, significant loss	6,445	6.1	
	Missing	1,248	1.2	
SARS-CoV-2 infection		379	0.4	
Being quarantined/isolated		19,560	18.5	
Pandemic-specific measures during the second period				
Income loss	No	82,077	77.5	
	Yes, some loss	16,268	15.4	
	Yes, significant loss	7,346	6.9	
	Missing	281	0.3	
SARS-CoV-2 infection		1,203	1.1	
Being quarantined/isolated		27,620	26.1	
Vaccinated (SARS-CoV-2)		2,182	2.1	
Pandemic-specific measures during the third period				
SARS-CoV-2 infection		2,405	2.3	
Being quarantined/isolated		8,463	8.0	
Vaccinated (SARS-CoV-2)		9,323	8.8	

BMI, Body Mass Index. N chronic medical conditions: Number of chronic medical conditions.

^a^ Values in % column indicate rate of non-missingness in the analytic sample for the first 9 rows, and the breakdown of the sample across groups thereafter.

### Predictors of Change in Mental Distress Levels at the Onset of the Pandemic

#### Baseline Sociodemographic and Health Characteristics

The associations of all covariates with initial distress level and the change across the three periods before and after adjusting for pre-pandemic level of distress are shown in Supplementary Tables S1, S2, respectively. Chronic medical conditions (see Supplementary Table S2 for parameter estimates), living alone (*β* = 0.28 [SE = 0.03]), female sex (*β* = 0.11 [0.01]), history of psychiatric disorders (*β* = 0.17 [0.01]), relative educational background (Supplementary Table S2), and obesity (*β* = 0.03 [0.01]) were associated with initial increases in mental distress (i.e., after adjustment for pre-pandemic mental distress). Additionally, being 35–44 years (relative to 45–54), was associated with a slight initial increase (*β* = 0.04 [0.01]).

**FIGURE 2:**
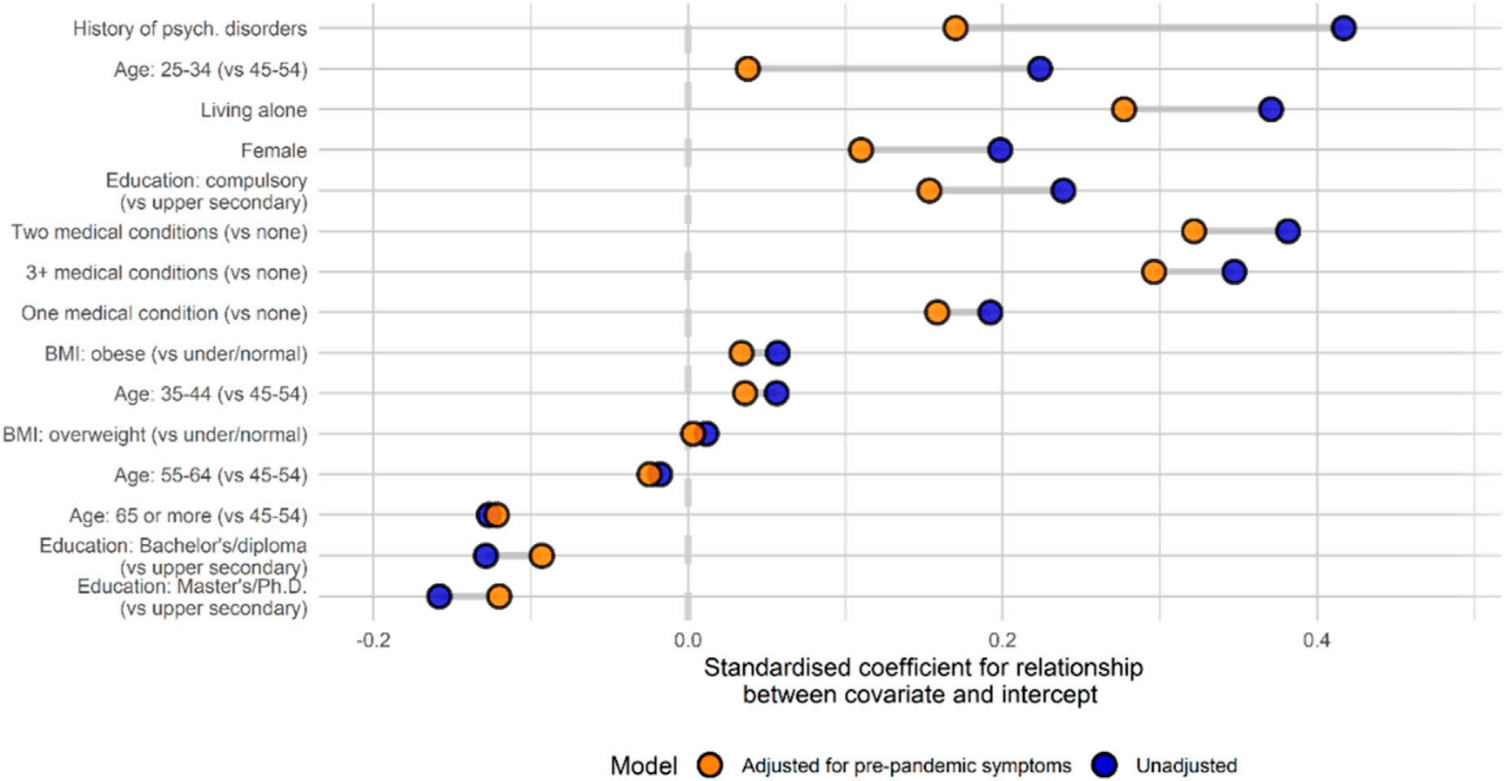
Associations between covariates and initial mental distress before and after adjusting for pre-pandemic mental distress (Oslo, Norway. 2023). Note: Covariates are ordered by change in coefficient between the two models. History of psych. Disorders: History of psychiatric disorders; BMI, Body Mass Index.

Figure 2 shows the differences in standardized association estimates between covariates and initial mental distress before and after adjusting for pre-pandemic mental distress. Virtually all effects were attenuated after adjustment for pre-pandemic mental distress. Effect sizes for history of mental disorders and younger age more than halved after adjustment, rendering the association between young age and initial changes in distress at the onset of the pandemic non-significant.

### Predictors of Change in Mental Distress Over the Course of the Pandemic

#### Baseline Sociodemographic and Health Characteristics

**FIGURE 3:**
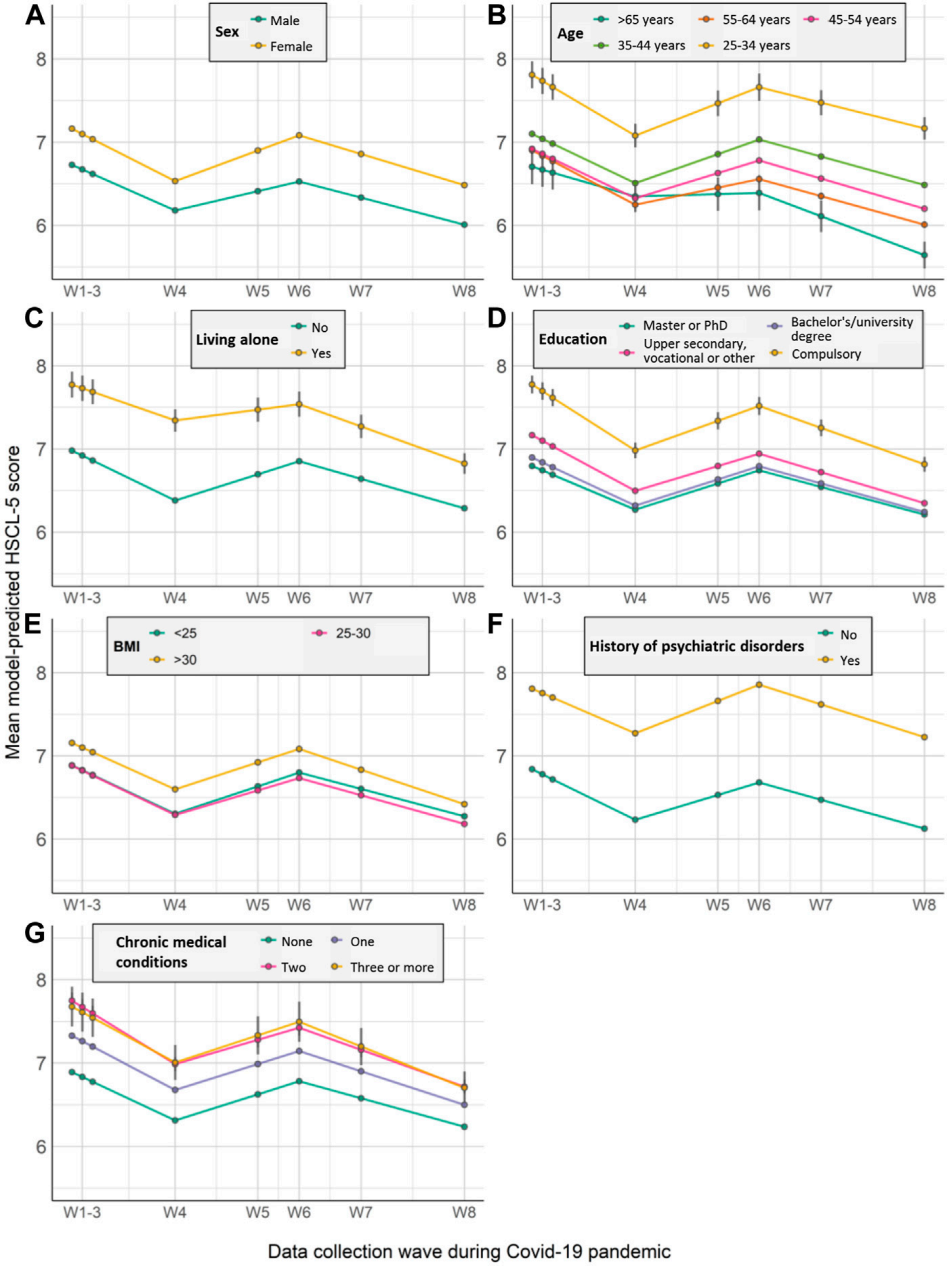
Model-predicted growth trajectories of estimated average mental distress score across waves of data collection (W1-W8) by participant characteristics **(A–G)** (Oslo, Norway. 2023). Notes: HSCL-5 = 5-item version of the Hopkins Symptoms Checklist. Error bar represents 95% confidence intervals; Medical comorbidities = Chronic medical conditions.

Figure 3 shows model-predicted mean HSCL scores stratified according to each baseline characteristic. Sex was the only factor influencing change across all three time periods, with females showing more rapid change in symptoms (*β*_1_ = −0.08 [0.01]; *β*_2_ = 0.14 [0.02]; *β*_3_ = −0.07 [0.01]). Change in symptoms during the first period was also associated with individuals’ educational background, with those with the highest education more likely to experience increases in symptoms (*β*_1_ = 0.13 [0.02]) and those with the lowest education more likely to experience decreases (*β*_1_ = −0.10 [0.04]). In addition, those with the highest BMI (*β*_1_ = 0.06 [0.02]), living alone (*β*_1_ = 0.14 [0.06]), and with a history of psychiatric disorders (*β*_1_ = 0.06 [0.02]) were also more likely to experience symptom increases during this period, while those with more chronic medical conditions were more likely to have reductions in symptom severity (having seen, on average, much greater increases at the onset of the pandemic; Supplementary Table S2). During the second period, the likelihood of experiencing reduced symptoms was associated with older age (particularly being older than 65; *β*_2_ = −0.28 [0.12]) and living alone (*β*_2_ = −0.21 [0.07]), reflecting a regression to the mean after prior increases in these groups. The likelihood of symptom increases continued to rise slightly for 34–44 years-olds (relative to those in the age band above; *β*_2_ = 0.04 [0.01]). The likelihood of symptom decreases during third period was associated with obesity (*β*_3_ = −0.09 [0.02]), having chronic medical conditions (Supplementary Table S2), again likely reflecting a reversion to the mean after earlier increases. Younger age continued to be associated with the likelihood of symptoms increasing—most notably among the youngest group (25–34 years; *β*_3_ = 0.16 [0.07]).

#### COVID-19-Related Factors

**FIGURE 4:**
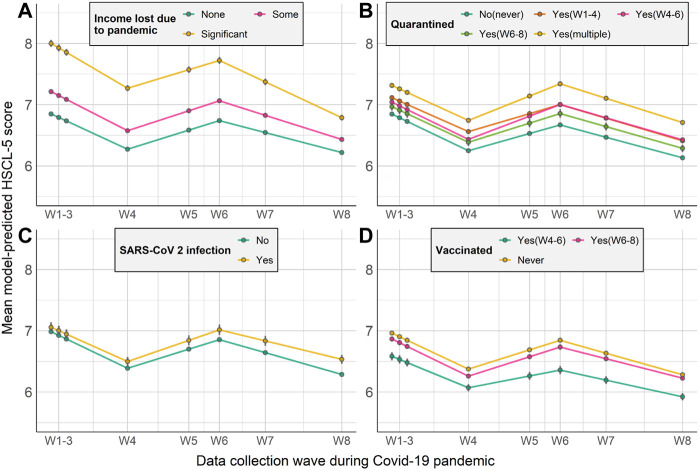
Model-predicted growth trajectories of estimated average mental distress score across waves of data collection (W1–W8) by COVID-19-related factors **(A–D)** (Oslo, Norway. 2023). Notes: HSCL-5 = 5-item version of the Hopkins Symptoms Checklist. Error bar represents 95% confidence intervals.

For the Covid-19-related factors (Figure 4), the likelihood of experiencing increases in mental distress in the first period was associated with significant income loss due to the pandemic in this period (*β*_1_ = 0.12 [0.02]), as well as being quarantined or having to isolate during this period (*β*_1_ = 0.08 [0.01]). During the second period, being vaccinated was associated with symptom reduction (*β*_2_ = −0.18 [0.03]) while symptom increases were again more likely among individuals quarantined during this period (*β*_2_ = 0.11 [0.01]). In the final period, those who suffered income loss during the prior period were more likely to see symptom reduction (*β*_3_ = −0.15 [0.02]), while SARS-CoV-2 infection was associated with likelihood of symptom increases (*β*_2_ = 0.10 [0.04]) for the first time (case numbers had remained very low in Norway during the two prior periods).

The effect of adjusting for pre-pandemic mental distress on estimated associations between covariates and change during each period are displayed in Supplementary Figures S3–S5.

## Discussion

Using prospectively collected data from a large nationwide cohort, we found that several factors were associated with initial increases in mental distress in the beginning of the pandemic. These included having chronic medical conditions or a history of mental disorders, relative lower education, female sex, living alone, and obesity, which were also associated with longitudinal change. Being quarantined and losing income due to COVID-19 were associated with likelihood of increasing distress during the first year of the pandemic. We found that having received the COVID-19 vaccination was associated with decreasing symptoms during the second period while SARS-CoV-2 infection was independently associated with increasing symptoms of mental distress only towards the end of the study period, which coincided with the first substantial rise in case numbers in Norway.

To isolate factors not just related to mental health in general, but to changes in mental health during the pandemic, we adjusted our models for pre-pandemic mental distress. A majority of studies regarding mental health and COVID-19 so far have been based on online recruitment and data collected after the start of the pandemic [7, 8, 27], without knowledge of pre-pandemic mental distress. We found the strength of associations with mental distress during the pandemic are reduced after adjustment for pre-pandemic data, particularly for variables such as age, history of psychiatric disorders, living alone, sex, education and chronic medical conditions. Findings from previous studies that did not control for these factors are likely to conflate general and specific effects on mental health and should be interpreted as such. To the extent that such findings are interpreted as being specific to the context of the pandemic, our results show that they are likely to be substantially inflated.

Our findings show that female sex and younger age were associated with higher initial level and more change in mental distress across time. This is in line with the results from a UK study performing growth models on anxiety symptoms and depressive symptoms [8]. It is documented that women are more likely to suffer from mental health problems than men [4, 9, 12, 14], and females had higher increase than males in prevalence of major depressive and anxiety disorders during COVID-19, particularly in younger age groups [14]. During the pandemic, additional carer and household responsibilities due to school closures or family members becoming unwell are more likely to fall on women [14, 28]. We also found that women demonstrated faster improvements in anxiety and depression symptoms during the first period.

Living alone was associated with both a higher initial level and less rapid recovery during the first period. Factors like living alone and loneliness have repeatedly been linked to mental health [12], also before the pandemic [29]. Interestingly, we observed that being older than 65 was associated with a relative reduction in risk for mental distress at the onset of, and during the pandemic. This was despite older age being a known risk factor for more severe disease from relatively early in the pandemic. Interacting sociodemographic factors, such as relative insulation from economic concerns or living in less densely populated areas and potential increase in social support may explain this somewhat counter-intuitive finding.

Individuals with chronic medical conditions presented with a substantial increase in mental distress at the initial stage of the pandemic, showed reversion towards the mean over time, similar to the UK study [8]. Obesity was predictive of increases in mental distress at the onset and for the early phase of the pandemic, and reversion toward the mean came later, during the period when vaccination became widespread. Obesity and chronic medical conditions are risk factors for COVID-19 severity [30], which was also communicated extensively during the first stages of the pandemic. This, followed by the implementation of greater shielding and infection control measures, as well as adjustment to perceived vulnerability, could explain the early increases and subsequent decreases in mental distress for these factors. Our findings of pre-existing mental illness as a significant predictor of mental problems were expected as it has been widely reported [3, 31, 32], although again we confirm that this is specific to the pandemic context by appropriately controlling for earlier symptoms. It is also worth noting that in contrast to medical comorbidities and obesity, individuals with pre-existing vulnerabilities to psychiatric disorders showed no reversion to the mean during our study period, suggesting that individuals with these vulnerabilities should be prioritised for support in analogous future situations.

COVID-19-related characteristics, such as loss of income and being quarantined, were associated with slower improvement during the first period. Economic loss is one significant adversity in the context of COVID-19, and could be where COVID-19 related stress partially originates from [33]. Being quarantined was associated with a greater likelihood of experiencing increases in symptoms during the second period. In addition to the obvious possibility that people were concerned that exposure to infected individuals might lead to them to contract the disease, poor housing quality and declining work performance due to lockdown during the early stage of the COVID-19 pandemic [34] might also explain these observations. Social isolation to mitigate the spread of the novel coronavirus may also have led to loneliness, which is associated with increased mental health problems [35]. During the second period, the likelihood of experiencing decreased symptoms was associated with being vaccinated. This protective effect of vaccination has been observed previously [36]. Decrease in mental distress symptoms during the third period was accentuated among those with significant income loss in the previous period. The greater improvement among those with income loss could be partially explained by the government mitigation strategies including unemployment benefit, i.e., more compensation would be offered under new rules [15]; and possible easing of economic and employment pressures. Individuals infected with SARS-CoV-2 were more likely to experience increased mental distress during the third period than those not infected. A negative association between SARS-CoV-2 infection and mental health have been observed in several studies [2, 3, 14] while our study extends the literature by robustly controlling for pre-pandemic levels of distress.

The results of this study should be interpreted in light of some limitations. First, the current study only included MoBa parents, therefore the results cannot necessarily be generalised to the adult population without children. The initial participation rate in MoBa was 41% of invited pregnant women, with previously described a strong underrepresentation of the youngest women (<25 years), those living alone, mothers with more than two previous births and with previous stillbirths [37] and an overrepresentation of healthier and highly educated women as compared to the general population [38]. In our sample, compared to age-adjusted population statistics from Statistics Norway for the year 2021, the most underrepresented groups were men aged 40–49 whose highest recorded level of education was either basic or upper secondary level. These individuals comprised 10.0% and 22.3% of the Norwegian male population in 2021 respectively, but only 1.0% and 7.8% of our analytic sample. 50–59 years-old men in the same educational categories were also substantially underrepresented, and in both cases individuals with completed vocational tertiary education or higher education were correspondingly over-sampled. Women were generally more representatively included in our analytic sample, but those aged 40–49 with the fewest completed years of education were again underrepresented (see Supplementary Table S3 for full comparison). These differences clearly limit the generalizability of the findings in some respects. Nonetheless, it should be highlighted that due to the size of the sample overall, individuals from underrepresented groups are still present in large numbers. Moreover, the assumption that effects are broadly linear and do not differ qualitatively in the extremes is not obviously an unrealistic one. Despite this, and given the apparently exacerbated impact of some aspects of the pandemic on those from poorer backgrounds, it is important the future work tests the wider applicability of these findings. A second limitation concerns our inability to draw causal conclusions about links between individual characteristics and trajectories of mental distress based on observational data. However, our findings extend current knowledge and provide important implications for future studies by taking the advantage of the longitudinal design and pre-pandemic information available in the MoBa cohort with data between the onset of the COVID-19 pandemic and majority vaccination against COVID-19.

In conclusion, our results identify several vulnerability factors, including living alone, being obese, history of psychiatric disorders that represent risk factors for both initial and longitudinal increases in mental distress in the context of the COVID-19 pandemic. Significant influences, too, of quarantine status, vaccination, and income loss due to the pandemic show that public health and governmental policies and priorities are likely to be influential at the level of individual mental health among citizens. These findings are important for planning of future public health responses to reduce the mental health impact of COVID-19 and similar future global health crises, as well as to optimize the allocation of health service resources and the design of prevention and intervention efforts.

## Data Availability

All analytic code is openly available online at https://github.com/psychgen/covid19-adult-mental-distress-trajectories. The consent given by the participants does not open for storage of data on an individual level in repositories or journals. Researchers who want access to datasets for replication should apply through helsedata.no.

## References

[1] Wiersinga WJ Rhodes A Cheng AC Peacock SJ Prescott HC. Pathophysiology, Transmission, Diagnosis, and Treatment of Coronavirus Disease 2019 (COVID-19): A Review. Jama (2020) 324(8):782–93. 10.1001/jama.2020.12839 32648899

[2] Magnúsdóttir I Lovik A Unnarsdóttir AB McCartney D Ask H Kõiv K Acute COVID-19 Severity and Mental Health Morbidity Trajectories in Patient Populations of Six Nations: An Observational Study. Lancet Public Health (2022) 7(5):e406–e416. 10.1016/S2468-2667(22)00042-1 35298894 PMC8920517

[3] Pierce M McManus S Hope H Hotopf M Ford T Hatch SL Mental Health Responses to the COVID-19 Pandemic: A Latent Class Trajectory Analysis Using Longitudinal UK Data. The lancet Psychiatry (2021) 8(7):610–9. 10.1016/S2215-0366(21)00151-6 33965057 PMC9764381

[4] Batterham PJ Calear AL McCallum SM Morse AR Banfield M Farrer LM Trajectories of Depression and Anxiety Symptoms During the COVID-19 Pandemic in a Representative Australian Adult Cohort. Med J Aust (2021) 214(10):462–8. 10.5694/mja2.51043 33899939 PMC8207103

[5] Taquet M Luciano S Geddes JR Harrison PJ. Bidirectional Associations Between COVID-19 and Psychiatric Disorder: Retrospective Cohort Studies of 62 354 COVID-19 Cases in the USA. The lancet Psychiatry (2021) 8(2):130–40. 10.1016/S2215-0366(20)30462-4 33181098 PMC7820108

[6] Zhou J Liu L Xue P Yang X Tang X. Mental Health Response to the COVID-19 Outbreak in China. Am J Psychiatry (2020) 177(7):574–5. 10.1176/appi.ajp.2020.20030304 32375540

[7] Shevlin M Butter S McBride O Murphy J Gibson-Miller J Hartman TK Refuting the Myth of a 'Tsunami' of Mental Ill-Health in Populations Affected by COVID-19: Evidence that Response to the Pandemic Is Heterogeneous, Not Homogeneous. Psychol Med (2021) 53(2):429–37. 10.1017/s0033291721001665 33875044 PMC8111207

[8] Fancourt D Steptoe A Bu F. Trajectories of Anxiety and Depressive Symptoms During Enforced Isolation Due to COVID-19 in England: A Longitudinal Observational Study. The lancet Psychiatry (2021) 8(2):141–9. 10.1016/S2215-0366(20)30482-X 33308420 PMC7820109

[9] Daly M Sutin AR Robinson E. Longitudinal Changes in Mental Health and the COVID-19 Pandemic: Evidence From the UK Household Longitudinal Study. Psychol Med (2020) 52:2549–58. 10.1017/S0033291720004432 33183370 PMC7737138

[10] Shanahan L Steinhoff A Bechtiger L Murray AL Nivette A Hepp U Emotional Distress in Young Adults During the COVID-19 Pandemic: Evidence of Risk and Resilience From a Longitudinal Cohort Study. Psychol Med (2020) 52:824–33. 10.1017/S003329172000241X 32571438 PMC7338432

[11] Hansen T Sevenius Nilsen T Knapstad M Skirbekk V Skogen J Vedaa Ø Covid-Fatigued? A Longitudinal Study of Norwegian Older Adults' Psychosocial Well-Being Before and During Early and Later Stages of the COVID-19 Pandemic. Eur J Ageing (2021) 19:463–73. 10.1007/s10433-021-00648-0 34456661 PMC8385264

[12] Bu F Steptoe A Fancourt D. Loneliness During a Strict Lockdown: Trajectories and Predictors During the COVID-19 Pandemic in 38,217 United Kingdom Adults. Soc Sci Med (2020) 265:113521. 10.1016/j.socscimed.2020.113521 33257177 PMC7768183

[13] Costanza A Macheret L Folliet A Amerio A Aguglia A Serafini G COVID-19 Related Fears of Patients Admitted to a Psychiatric Emergency Department During and Post-Lockdown in Switzerland: Preliminary Findings to Look Ahead for Tailored Preventive Mental Health Strategies. Medicina (Kaunas) (2021) 57(12):1360. 10.3390/medicina57121360 34946305 PMC8707997

[14] COVID-19 Mental Disorders Collaborators. Global Prevalence and Burden of Depressive and Anxiety Disorders in 204 Countries and Territories in 2020 Due to the COVID-19 Pandemic. Lancet (2021) 398:1700–12. 10.1016/S0140-6736(21)02143-7 34634250 PMC8500697

[15] The Norwegian Government. Timeline: News From Norwegian Ministries About the Coronavirus Disease Covid-19 (2022). Available From: https://www.regjeringen.no/en/topics/koronavirus-covid-19/timeline-for-news-from-norwegian-ministries-about-the-coronavirus-disease-covid-19/id2692402/ (Accessed December 7, 2021).

[16] Salanti G Peter N Tonia T Holloway A White IR Darwish L The Impact of the COVID-19 Pandemic and Associated Control Measures on the Mental Health of the General Population: A Systematic Review and Dose-Response Meta-Analysis. Ann Intern Med (2022) 175(11):1560–71. 10.7326/M22-1507 36252247 PMC9579966

[17] Magnus P Birke C Vejrup K Haugan A Alsaker E Daltveit AK Cohort Profile Update: The Norwegian Mother and Child Cohort Study (MoBa). Int J Epidemiol (2016) 45(2):382–8. 10.1093/ije/dyw029 27063603

[18] Magnus P Irgens LM Haug K Nystad W Skjaerven R Stoltenberg C Cohort Profile: The Norwegian Mother and Child Cohort Study (MoBa). Int J Epidemiol (2006) 35(5):1146–50. 10.1093/ije/dyl170 16926217

[19] Tambs K Moum T. How Well Can a Few Questionnaire Items Indicate Anxiety and Depression? Acta Psychiatr Scand (1993) 87(5):364–7. 10.1111/j.1600-0447.1993.tb03388.x 8517178

[20] Schmalbach B Zenger M Tibubos AN Kliem S Petrowski K Brähler E. Psychometric Properties of Two Brief Versions of the Hopkins Symptom Checklist: HSCL-5 and HSCL-10. Assessment (2021) 28(2):617–31. 10.1177/1073191119860910 31272193

[21] Strand BH Dalgard OS Tambs K Rognerud M. Measuring the Mental Health Status of the Norwegian Population: A Comparison of the Instruments SCL-25, SCL-10, SCL-5 and MHI-5 (SF-36). Nord J Psychiatry (2003) 57(2):113–8. 10.1080/08039480310000932 12745773

[22] Rosseel Y. Lavaan: An R Package for Structural Equation Modeling. J Stat Softw (2012) 48(2):1–36. 10.18637/jss.v048.i02

[23] R Core Team. R: A Language and Environment for Statistical Computing. Vienna, Austria: R Foundation for Statistical Computing (2020). Avaialable From: https://www.R-project.org/ (Accessed August 28, 2023).

[24] RStudio Team. RStudio: Integrated Development for R. RStudio, Inc. Boston, MA: RStudio Team (2020). Avaialable From: http://wwwrstudiocom/ (Accessed August 28, 2023).

[25] Enders CK Bandalos DL. The Relative Performance of Full Information Maximum Likelihood Estimation for Missing Data in Structural Equation Models. Struct equation Model (2001) 8(3):430–57. 10.1207/s15328007sem0803_5

[26] Hu LT Bentler PM. Cutoff Criteria for Fit Indexes in Covariance Structure Analysis: Conventional Criteria Versus New Alternatives. Struct equation Model a multidisciplinary J (1999) 6(1):1–55. 10.1080/10705519909540118

[27] Saunders R Buckman JEJ Fonagy P Fancourt D. Understanding Different Trajectories of Mental Health Across the General Population During the COVID-19 Pandemic. Psychol Med (2021) 52:4049–57. 10.1017/S0033291721000957 PMC8007951 33653426

[28] Burki T. The Indirect Impact of COVID-19 on Women. Lancet Infect Dis (2020) 20(8):904–5. 10.1016/S1473-3099(20)30568-5 32738239 PMC7836874

[29] Coyle CE Dugan E. Social Isolation, Loneliness and Health Among Older Adults. J Aging Health (2012) 24(8):1346–63. 10.1177/0898264312460275 23006425

[30] Gao F Zheng KI Wang XB Sun QF Pan KH Wang TY Obesity Is a Risk Factor for Greater COVID-19 Severity. Diabetes Care (2020) 43(7):e72–e4. 10.2337/dc20-0682 32409499

[31] Breslau N Roth T Rosenthal L Andreski P. Sleep Disturbance and Psychiatric Disorders: A Longitudinal Epidemiological Study of Young Adults. Biol Psychiatry (1996) 39(6):411–8. 10.1016/0006-3223(95)00188-3 8679786

[32] North CS Nixon SJ Shariat S Mallonee S McMillen JC Spitznagel EL Psychiatric Disorders Among Survivors of the Oklahoma City Bombing. Jama (1999) 282(8):755–62. 10.1001/jama.282.8.755 10463711

[33] Pfefferbaum B North CS. Mental Health and the Covid-19 Pandemic. New Engl J Med (2020) 383(6):510–2. 10.1056/NEJMp2008017 32283003

[34] Amerio A Bertuccio P Santi F Bianchi D Brambilla A Morganti A Gender Differences in COVID-19 Lockdown Impact on Mental Health of Undergraduate Students. Front Psychiatry (2021) 12:813130. 10.3389/fpsyt.2021.813130 35069298 PMC8766745

[35] Killgore WDS Cloonan SA Taylor EC Dailey NS. Loneliness: A Signature Mental Health Concern in the Era of COVID-19. Psychiatry Res (2020) 290:113117. 10.1016/j.psychres.2020.113117 32480121 PMC7255345

[36] Xiao Y Yip PS Pathak J Mann JJ. Association of Social Determinants of Health and Vaccinations With Child Mental Health During the COVID-19 Pandemic in the US. JAMA Psychiatry (2022) 79(6):610–21. 10.1001/jamapsychiatry.2022.0818 35475851 PMC9047762

[37] Nilsen RM Vollset SE Gjessing HK Skjaerven R Melve KK Schreuder P Self-Selection and Bias in a Large Prospective Pregnancy Cohort in Norway. Paediatr Perinat Epidemiol (2009) 23(6):597–608. 10.1111/j.1365-3016.2009.01062.x 19840297

[38] Bulik CM Von Holle A Hamer R Knoph Berg C Torgersen L Magnus P Patterns of Remission, Continuation and Incidence of Broadly Defined Eating Disorders During Early Pregnancy in the Norwegian Mother and Child Cohort Study (MoBa). Psychol Med (2007) 37(8):1109–18. 10.1017/S0033291707000724 17493296 PMC2657803

